# Synthesis and Biological Activity of New [1,3]Thiazolo[4,5-*d*]pyridazin-4(5*H*)-ones

**DOI:** 10.3797/scipharm.1505-16

**Published:** 2015-08-27

**Authors:** Anatoly Demchenko, Ludmila Bobkova, Oleh Yadlovskiy, Tatiana Buchtiarova, Sergey Demchenko

**Affiliations:** Institute of Pharmacology and Toxicology, Eugene Pottier 14, 03057 Kiev, Ukraine

**Keywords:** [1,3]Thiazolo[4,5-*d*]pyridazinones, Anti-inflammatory activity, Analgesic activity, *In vivo* tests, «Hot plate» model, «Acetic acid cramps» model

## Abstract

A series of novel 2-(*N*-pyrrolidino, *N*-piperidino or *N*-morpholino)-7-phenyl(α-furoyl or α-thienyl)-[1,3]thiazolo[4,5-*d*]pyridazinones **10a–c, 14–16a,b** was synthesized in 78–87% yields via the reaction of methyl 5-benzoyl(α-furoyl or α-thienyl)-2-aminosubstituted-thiazol-4-carboxylates **9a–c, 13a–e** with hydrazine. These new compounds have been tested for their *in vivo* analgesic and anti-inflammatory activities. All compounds have been characterized by ^1^H-NMR, ^13^C-NMR spectroscopy, and liquid chromatography–mass spectrometry.

## Introduction

Synthesis of novel 7-Chloro-4-({[5-(6,8-dibromo-2-oxo-2*H*-1-benzopyran-3-yl)-1,3-thiazol-2-yl]amino}methyl)quinolin-2(1*H*)-one as an anti-inflammatory and analgesic agent that contains a thiazole ring was described previously [1]. It has been reported that RS-57067 is a selective COX-2 inhibitor [2]. The anti-inflammatory and analgesic activity was shown in compounds containing the thiazole and pyridazinone fragments (Sch. 1). The combination of the 2-aminothiazole and pyridazinone fragments can lead to interesting anti-inflammatory properties and analgesic activities.

**Sch. 1:**
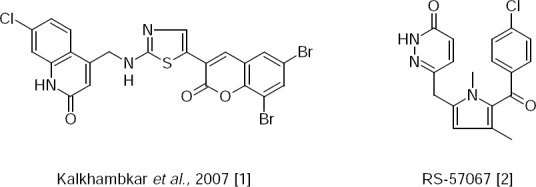
Some selected models of 2-aminothiazole and the pyridazinone derivatives possessing anti-inflammatory activity.

## Results and Discussion

### Chemistry

Intermediate compounds **4a–c** for the synthesis of new [1,3]thiazolo[4,5-*d*]pyridazinones were synthesized by Scheme 2.

**Sch. 2:**
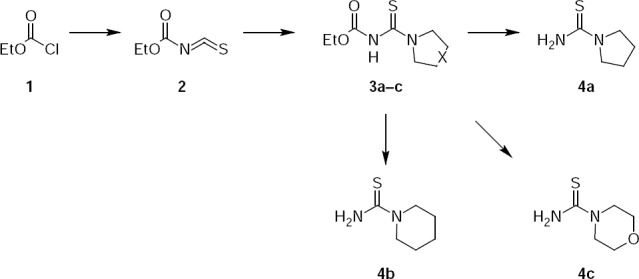
Synthetic routes for the preparation of compounds 4a–c.

1-Pyrrolidinecarbothioamide **4a** was obtained with the yield 73% through the condensation of pyrrolidine with ethyl isothiocyanatidocarbonate **2** and further hydrolysis of ester **3** with a well-known technique [3]. Key ethyl isothiocyanatidocarbonate **2** was synthesized by a modified technique [4] from ethyl chloroformate and dry potassium rhodanide in the presence of tetramethylethylenediamine (TMEDA) as a catalyst.

1-Piperidinecarbothioamide **4b** and 4-morpholinecarbothioamide **4c** were synthesized by a method similar to 1-pyrrolidinecarbothioamide **4a**. Constants and spectral characteristics of compounds **4b,c** correspond to literature data [5, 6].

7-Phenyl-2-(1-pyrrolidinyl)thiazolo[4,5-*d*]pyridazin-4(5*H*)-one **10a** was synthesized by Scheme 3.

**Sch. 3:**
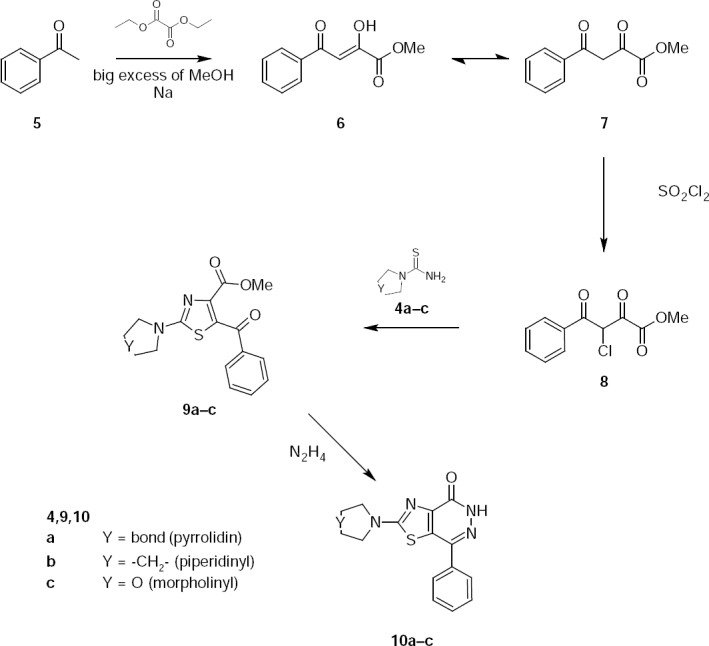
Synthesis of 2-substituted 7-phenyl-thiazolo[4,5-*d*]pyridazin-4(5*H*)-ones.

At the condensation of acetophenone with ethyl oxalate in the presence of sodium methylate is formed 2,4-dioxobenzenebutanoic acid methyl ester **7** [7]. Ester **7** was chlorinated by sulfuryl chloride in boiling, dry chloroform to give 3-chloro-2,4-dioxo-4-phenylbutanoic acid methyl ester **8** [8]. Under the conditions of the Ganch reaction [9], ester **8** reacts with 1-pyrrolidinecarbothioamide **4a** and **9a** is obtained. By refluxing **9a** with hydrazine hydrate in ethanolic solution, 7-phenyl-2-(1-pyrrolidinyl)-thiazolo[4,5-*d*]pyridazin-4(5*H*)-one **10a** is isolated in high yield.

1-Piperidinecarbothioamide **4b** and 4-morpholinecarbothioamide **4c** react with ester **8** in benzene that leads to the formation of the corresponding thiazoles **9b,c**, which were refluxed with hydrazine hydrate in ethanol. In this way, 7-phenyl-2-(1-piperidinyl)-thiazolo[4,5-*d*]pyridazin-4(5*H*)-one **10b** and 2-(4-morpholinyl)-7-phenyl-thiazolo[4,5-*d*]pyridazin-4(5*H*)-one **10c** were synthesized.

**Sch. 4:**
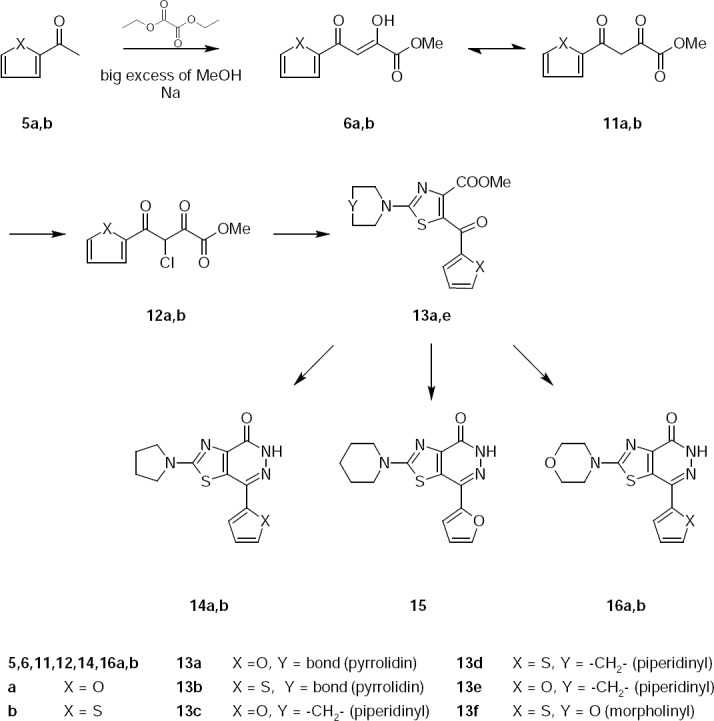
Synthesis of 2-substituted 7-heteryl-thiazolo[4,5-*d*]pyridazin-4(5*H*)-one.

4-Furan-2-yl-2,4-dioxobutyric acid methyl ester 11a and 2,4-dioxo-4-thiophen-2-yl-butyric acid methyl ester 11b were synthesized from 2-acetylfuran 5a and 2-acetylthiophene 5b by the reaction with ethyl oxalate in the presence of sodium methylate by method [10,11].

Methyl esters 3-chloro-4-furan(thiophen)-2-yl-2,4-dioxo-butyric acid 12a,b were used in further reactions without isolation or purification.

Reaction of esters 12a,b with 1-pyrrolidinecarbothioamide 4a–c led to the formation of methyl esters 13a–e, which were not isolated from the reaction mixture. 7-Furan(thiophen)-thiazolo[4,5-*d*]pyridazin-4(5*H*)-ones 14a,b, 15, and 16a,b were synthesized by refluxing of the corresponding esters 13a–e with double excess of hydrazine hydrate.

The synthesized novel 2-(*N*-pyrrolidino, *N*-piperidino or *N*-morpholino)-7-phenyl(α-furoyl or α-thienyl)-[1,3]thiazolo[4,5-*d*]pyridazinones 10a–c, 14a,b, 15, and 16a,b were characterized by ^1^H- and ^13^C-NMR and elemental analyses (see “Experimental” section).

In the ^1^H-NMR spectra of methyl esters 9a-c and 13a-e, singlet signals of methyl ester groups were registered in the region 3.78–3.95 ppm. Signals of the pyrrolidine substituent of compounds 9a, 13a, and 13b were observed in the form of two broad triplets – -CH_2_-CH_2_- at 2.05–2.08 ppm and -CH_2_NCH_2_- at 3.55–3.58 ppm, correspondingly. Signals of the piperidine substituents of compounds 9b and 13c were registered in a form of six proton multiplets – -CH_2_-CH_2_-CH_2_- at 1.65–1.73 ppm and four proton multiplets – -CH_2_NCH_2_- at 3.56 ppm. Signals of the morpholine fragment of compounds 9c, 13d, and 13e were observed in the form of four broad proton triplets – -CH_2_NCH_2_- at 3.63–3.66 ppm and -CH_2_OCH_2_- at 3.76-3.77 ppm, correspondingly.

After the condensation of esters 9a–c and 13a–e to [1,3]thiazolo[4,5-*d*]pyridazinones 10a–c, 14–16a,b in the ^1^H-NMR spectra, signals of the methyl ester group were not observed and appeared as broad singlet signals of the NH proton at 12.8–13.1 ppm. These signals disappeared after adding one drop of D_2_O to the sample before recording the spectra.

Signals of the protons of furan and thiophene substituents in compounds 13a–e after condensation into [1,3]thiazolo[4,5-*d*]pyridazinones 10a–c, 14–16a,b changed very little. For example, after condensation of 5-(furan-2-carbonyl)-2-pyrrolidin-1-yl-thiazole-4-carboxylic acid methyl ester (13a) to 7-furan-2-yl-2-pyrrolidin-1-yl-5H-thiazolo[4,5-*d*]pyridazin-4-one (14a), one proton doublet of the proton in 3rd position of the furane cycle was observed at 6.94 ppm for 13a and at 7.01 ppm for 14a, one proton multiplet in 4th position was observed at 6.65 ppm for 13a and at 6.68 ppm for 14a; one proton doublet in 5th position was registered at 7.85 ppm for 13a and at 7.89 ppm for 14a. Analogous changes were observed for thiophene-substituted compounds 13b and 14b – one proton doublet at 3rd position: 7.66 ppm and 7.54 ppm, correspondingly, one proton triplet at 4th position: 7.24 ppm and 7.20 ppm, correspondingly, and one proton doublet: 7.85 ppm and 7.67 ppm, correspondingly.

### Pharmacology

Analgesics that are used today, whose mechanism of action is associated with the effect on COX isoenzymes, have significant side effects. Therefore, it is important to search new analgesics exceeding the existing ones in efficacy and/or safety [12]. This study was conducted to examine the possible peripheral and central action of new analgesics. The study is a fragment of the determination of the efficiency derivatives of 2-(*N*-pyrrolidino, *N*-piperidino or *N*-morpholino)-7-phenyl(α-furoyl or α-thienyl)-[1,3]thiazolo[4,5-*d*]pyridazinones in acute pain. Pain syndrome that occurs after intraperitoneal injection of acetic acid in mice is manifested through a peculiar abdominal constriction (writhing). It is associated with irritation of the peritoneum and is regarded as a model of visceral pain. In this case, intraperitoneal injection of 0.6% acetic acid solution assists in a general activation of the nociceptive system and local release of bradykinin, histamine, serotonin, prostaglandins, etc [13]. Acetic acid causes an increase in peritoneal fluid levels of prostaglandins (PGE2 and PGF2α), involving in part peritoneal receptors and inflammatory pain by inducing capillary permeability [14, 15]. The above result indicates that in the model of nociceptive stimulation (writhing caused by intraperitoneal injection of acetic acid), the substance out-performs ketorolac by about 1-fold.

The hot plate model describes the supraspinal level of nociception, which may allow us to define the central component of the antinociceptive activity of the analgesic. This test characterizes the effectiveness of the compound as to its suppression of somatic superficial and acute pain. These data indicate a central component of analgesia derivatives of [1,3]thiazolo[4,5-*d*]pyridazinones (**10b, 10c, 14b, 16a, 16b**).

The substances **10a** and **16a** have shown an antiexudative effect (30.97% and 34.8%, respectively) in the carrageenan edema model on mice at the dose 25 mg/kg (Table 1).

**Tab. 1:**
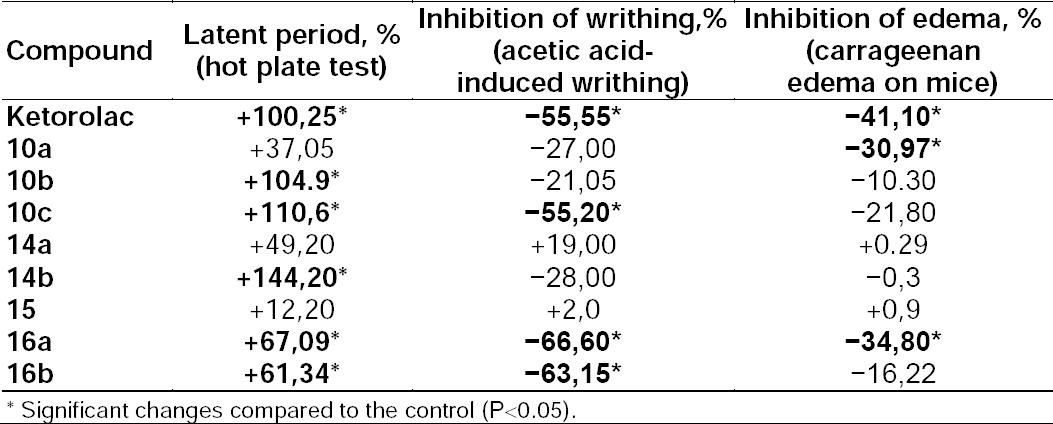
Comparative evaluation of analgesic activity and antiexudative effect of derivatives [1,3]thiazolo[4,5-*d*]pyridazinones (**10a–c**, **14–16a,b**) and ketorolac

The reference medicine ketorolac induced a 41.1% edema reduction at the study dose as compared to the control group. In applying substances **10a, 16a,b** in the acetic acid-induced writhing test on the mice at the dose of 25 mg/kg, a significant analgesic effect was determined 60 min after administration (Table 1).

For this purpose, the study substance and ketorolac as the reference drug were administered 60 min before injection of acetic acid and the amount of writhing in the control and treated groups was recorded**.** It was found that in the dose of 25 mg/kg, the compounds inhibited the amount of writhing in the range from −21.05% to −66.60%.

In the hot plate test, the five derivatives of [1,3]thiazolo[4,5-*d*]pyridazinones (**10b,c, 14b, 16a,b**) showed a significant analgesic effect (P>0.05) in doses of 25 mg/kg (Table 1). The substance **14b** was more effective than the reference drug ketorolac.

The basic structures with the phenyl substituent in position 7 (**10b,c**) provided the antinociceptive impact close to that caused by 104.9–110.6% ketorolac on the “hot plate” model which indicates the central component of the analgesic influence. Substituting the nitrogen atom for the oxygen atom in the pyridine fragment modulated the pronounced central and peripheral components of pain (**10a–c**).

Compound **15**, with a furane ring and a piperidine fragment in its structure (the systems’ second position), manifested no analgesic or anti-inflammatory activities: +12.2% (“hot plate”); +2.0% (acetous “writhing”); +0.9% (carrageen paw edema).

Substituting the piperidine fragment for the morpholine ring resulted in the growth of antinociceptive activity on the “hot plate” models (+67.09% and +61.34%) as well as acetous “writhing” (−66.60% and −63.15%) for compounds **16a,b**, respectively.

Substituting the oxygen atom for a sulfur atom within the furane ring provided no sufficient effect.

Respective substitution of the piperidine ring for a pyrrole fragment and the furane ring for a thiophene fragment (**14b**) allowed registering the biggest value of the antinociceptive effect +144.2% on the “hot plate”. On the contrary, substituting the thiophene fragment for the furane fragment cancelled (**14a**) the antinociceptive effect +49.2%.

Anti-inflammatory activity of the studied compounds was low and thus, was inferior to ketolac to which they were compared. Out of the basic structures with the phenyl substitutor in position 7 (**10a–c**), the **10a** structure was the only one to reveal a valid antiexudative effect (−30.97%). Meanwhile, this structure was not active in the acetous “writhing” model where mediators of inflammation participate in generating nociceptive sensation. The **16a** compound revealed valid antiexudative activity as well as antinociceptive (−34.8%). Therefore, we can regard it as a starting point to search further for non-steroidal anti-inflammatory drugs.

## Conclusion

In conclusion, we synthesized a series of new [1,3]thiazolo[4,5-*d*]pyridazinones. Comparative evaluations of analgesic activity and the antiexudative effect of derivatives [1,3]thiazolo[4,5-*d*]pyridazinones with ketorolac on the «hot plate» and «acetic acid cramps» models were studied. Compounds exceeding the analgesic activity of ketorolac were found. It was shown that the compounds of this series exhibited moderate anti-inflammatory activity.

## Experimental

### Chemistry

All solvents were purified before use. Ethyl chlorophormiate, pyrrolidine, morpholine, piperidine, acetophenone, ethyl oxalate, *2*-acetylthiophene, and *2*-acetylfurane were purchased from Acros Organics and used without purification. Reactions were monitored by thin-layer chromatography (TLC) using Fluka silica gel (60 F 254) plates (0.25 mm). Visualization was made with UV light. Melting points of the synthesized compounds were taken on a melting point tube. ^1^H-NMR spectra were recorded on the Varian Gemini 400 MHz (Germany) in DMSO-d6 using tetramethylsilane (TMS) as an internal standard. Chemical shifts are reported in ppm units with use of the d scale. The mass spectra were recorded on an Agilent LC/MSD SL 1100 instrument (USA).

### General Procedure of Synthesis of 2-(RR_1_-amino)-5-benzoyl(α-furoyl or α-thienoyl)-thiazole-4-carboxylic acid methyl esters 9a–c, 13a–e.

A mixture of an appropriate amount of 3-chloro-2,4-dioxo-4-benzenebutanoic acid methyl ester **8** or 3-chloro-4-furan/thiophen-2-yl-2,4-dioxobutyric acid methyl ester **12a,b** and (0.01 mole) 1-pyrrolidine(or piperidine)carbothioamide **4a,b (**4-morpholinecarbothioamide **4c**) was refluxed for 4 h in 50 ml of methanol and left overnight at room temperature. The reaction mixture was diluted with 200 ml of distilled water and a 10% solution of NaOH was added till pH 8. The obtained solid products were collected by filtration, washed with water, and recrystallized from the appropriate solvent.

### Methyl 5-benzoyl-2-(pyrrolidin-1-yl)-1,3-thiazole-4-carboxylate (9a)

Yield 85%, mp 165–167°C (MeOH). ^1^H-NMR (400 MHz, DMSO-d_6_) δ: 2.06 (t, 4H, -CH_2_-CH_2_-), 3.56 (t, 4H-CH_2_NCH_2_-), 3.88 (s, 3H, COOCH_3_), 7.45 (t, 2H, Ph), 7.61 (t, 1H, Ph), 7,99 (d, 2H, Ph). Anal. calcd for C_16_H_16_N_2_O_3_S, % N, 8.85; S 10.1. Found, % N 8.66; S 9.95.

### Methyl 5-benzoyl-2-(piperidin-1-yl)-1,3-thiazole-4-carboxylate (9b)

Yield 83%, mp 138–139°C (MeOH). ^1^H-NMR (400 MHz, DMSO-d_6_) δ: 1.65-1.73 (m, 6H, -CH_2_-CH_2_-CH_2_-), 3.56 (m, 4H, -CH_2_NCH_2_-), 3.78 (s, 3H, COOCH_3_), 7.47 (t, 2H, Ph), 7.56 (t, 1H, Ph), 8,01 (d, 2H, Ph). Anal. calcd for C_17_H_18_N_2_O_3_S, % N, 8.48; S 9.70. Found,% N 8.65; S 9.61.

### Methyl 5-benzoyl-2-(morpholin-1-yl)-1,3-thiazole-4-carboxylate (9c)

Yield 81%, mp 121–123°C (MeOH). ^1^H-NMR (400 MHz, DMSO-d_6_) δ: 3.63 (t, 4H, -CH_2_NCH_2_-), 3.77 (t, 4H, -CH_2_OCH_2_-), 3.95 (s, 3H, COOCH_3_), 7.46 (t, 2H, Ph), 7.59 (t, 1H, Ph), 8,00 (d, 2H, Ph). Anal. calcd for C_16_H_16_N_2_O_4_S, %N 8.42; S 9.63. Found,% N 8.29; S 9.42.

### Methyl 5-(2-furoyl)-2-(pyrrolidin-1-yl)-1,3-thiazole-4-carboxylate (13a)

Yield 76%, mp 135–136°C (MeOH). ^1^H-NMR (400 MHz, DMSO-d_6_) δ: 2.08 (t, 4H, -CH_2_-CH_2_-), 3.58 (t, 4H, -CH_2_NCH_2_-), 3.81 (s, 3H, OCH_3_), 6.65 (m,1H, 4^1^H), 6.94 (d, 1H, 3^1^H), 7.85 (d, 1H, 5^1^H). Anal. calcd for C_14_H_14_N_2_O_4_S, % N 9.14; S 10.50. Found, % N 9.10; S 10.4.

### Methyl 2-(pyrrolidin-1-yl)-5-(2-thienylcarbonyl)-1,3-thiazole-4-carboxylate (13b)

Yield 81%, mp 161–162°C (MeOH). ^1^H-NMR (400 MHz, DMSO-d_6_) δ: 2.05 (t, 4H, -CH_2_-CH_2_-), 3.55 (t, 4H, -CH_2_NCH_2_-), 3.79 (s, 3H, COOCH_3_), 7,24 (t, 1H, 4^1^H), 7,66 (d, 1H, 3^1^H), 7,85 (d,1H, 5^1^H). Anal. calcd for C_14_H_14_N_2_O_3_S_2_, % N 8.69; S 19.9. Found, % N 8.44; S 19.6.

### Methyl 5-(2-furoyl)-2-(piperidin-1-yl)-1,3-thiazole-4-carboxylate (13c)

Yield 78%, mp 145–147°C (MeOH). ^1^H-NMR (400 MHz, DMSO-d_6_) δ: 1.68-1.72 (m, 6H, -CH_2_-CH_2_-CH_2_-), 3.56 (m, 4H, -CH_2_NCH_2_-), 3.85 (s, 3H, COOCH_3_), 6.68 (t, 1H, 4^1^H), 6.95 (d, 1H, 3^1^H), 7.83 (d, 1H, 5^1^H). Anal. calcd for C_15_H_16_N_2_O_4_S, % N 8.74; S 10.00. Found, % N 8.65; S 10.20.

### Methyl 5-(2-furoyl)-2-(morpholin-4-yl)-1,3-thiazole-4-carboxylate (13d)

Yield 85%, mp 163–164°C (MeOH). ^1^H-NMR (400 MHz, DMSO-d_6_) δ: 3.64 (t, 4H, -CH_2_NCH_2_-), 3.76 (t, 4H, -CH_2_OCH_2_-), 3.94 (s,3H, COOCH_3_), 6.67 (t, 1H, 4^1^H), 6.93 (d, 1H, 3^1^H), 7.85 (d, 1H, 5^1^H). Anal. calcd for C_14_H_14_N_2_O_5_S, % N 8.69; S 9.95. Found, % N 8.66; S 9.87.

### Methyl 2-(morpholin-4-yl)-5-(2-thienylcarbonyl)-1,3-thiazole-4-carboxylate (13e).

Yield 87%, mp 155–156°C (MeOH). ^1^H-NMR (400 MHz, DMSO-d_6_) δ: 3.66 (t, 4H, -CH_2_NCH_2_-), 3.77 (t, 4H, -CH_2_OCH_2_-), 3.86 (s,3H, COOCH_3_), 7,23 (t, 1H, 4^1^H), 7,67 (d, 1H, 3^1^H), 7,83 (d, 1H, 5^1^H). Anal. calcd for C_14_H_14_N_2_O_4_S_2_, % N 8.28; S 19.0. Found, % N 8.23; S 18.9.

### General Procedure of Synthesis of 2-(RR_1_-Amino)-7-phenyl(furan-2-yl or thiophen-2-yl)-5H-thiazolo[4,5-d]pyridazin-4-ones 10a–c, 14–16a,b.

0.01 mole of the corresponding compounds **9a–c**, **13a–e** were suspended in 100 ml of ethanol, a two-fold excess of hydrazine hydrate was added to the mixture. The mixture was refluxed for 4 hours. The reaction mixture was cooled. The precipitate was filtered off and crystallized from a mixture of ethanol-DMF (1:1).

### 7-Phenyl-2-(pyrrolidin-1-yl)[1,3]thiazolo[4,5-d]pyridazin-4(5H)-one (10a)

Yield 77%, mp 285–286°C, ^1^H-NMR (400 MHz, DMSO-d_6_), δ: 2.05 (s,4H, -CH_2_-CH_2_-), 3.54 (s, 4H, -CH_2_NCH_2_-), 7.47–7.56 (m, 3H, Ph), 7.75–7.77 (m, 2H, Ph), 12.7 (s, 1H, NH). Anal. calcd for C_15_H_14_N_4_OS, % N 18.8; S 10.8. Found, % N 18.6; S 10.9. MS m/z: 299.1 [(M+H)+].

### 7-Phenyl-2-(piperidin-1-yl)[1,3]thiazolo[4,5-d]pyridazin-4(5H)-one (10b).

Yield 78%, mp 308–309°C, ^1^H-NMR (400 MHz, DMSO-d_6_), δ: 1.63 (m, 6H, -CH_2_-CH_2_-CH_2_-), 3.61 (m, 4H, -CH_2_NCH_2_-), 7.49 – 7.55 (m, 3H, Ph), 7.73 – 7.75 (m, 2H, Ph), 13.0 (s, 1H, NH). ^13^C-NMR (100 MHz, DMSO-d_6_) δ: 23.4, 24.8, 49.5, 126.7, 128.9, 129.3, 135.6, 135.7, 140.5, 140.6, 148.4, 155.2, 170.9. Anal. calcd for C_16_H_16_N_4_OS, % N 17.9; S 10.3. Found, % N 17.8; S 10.4. MS m/z: 313.1 [(M+H)+].

### 2-(Morpholin-4-yl)-7-phenyl[1,3]thiazolo[4,5-d]pyridazin-4(5H)-one (10c).

Yield 81%, mp 302–303°C, ^1^H-NMR (400 MHz, DMSO-d_6_), δ: 3.62 (t,4H, -CH_2_NCH_2_-), 3.74 (t, 4H, -CH_2_OCH_2_-), 7.51–7.57 (m, 3H, Ph), 7.74 – 7.76 (m, 2H, Ph), 13.1 (s, 1H, NH). ^13^C-NMR (100 MHz, DMSO-d_6_) δ: 48.3, 65.2, 126.7, 127.6, 128.9, 129.4, 135.6, 140.6, 148.1, 155.2, 171.4. Anal. calcd for C_15_H_14_N_4_O_2_S, % N 17.8; S 10.2. Found, % N 17.7; S 10.4. MS m/z: 315.0 [(M+H)+].

### 7-(Furan-2-yl)-2-(pyrrolidin-1-yl)[1,3]thiazolo[4,5-d]pyridazin-4(5H)-one (14a)

Yield 83%, mp 292–293°C, ^1^H-NMR (400 MHz, DMSO-d_6_), δ: 2.04 (m, 4H, -CH_2_-CH_2_-), 3.50 (m, 4H, -CH_2_NCH_2_-), 6.70 (m, 1H, 4^1^H), 6.96 (d, 1H, 3^1^H), 7.92 (d, 1H, 5^1^H), 12.9 (s, 1H, NH). ^13^C-NMR (100 MHz, DMSO-d_6_) δ: 25.2, 49.9, 108.2, 112.4, 144.0, 155.2, 155.3, 168.2, 193.2. Anal. calcd for C_13_H_12_N_4_O_2_S, % N 19.4; S 11.1. Found, % N 19.1; S 11.2. MS m/z: 289.1 [(M+H)+].

### 2-(Pyrrolidin-1-yl)-7-(thiophen-2-yl)[1,3]thiazolo[4,5-d]pyridazin-4(5H)-one (14b)

Yield 81%, mp 320–322°C, ^1^H-NMR (400 MHz, DMSO-d_6_), δ: 2.07 (t, 4H, -CH_2_-CH_2_-), 3.58 (t, 4H, -CH_2_NCH_2_-), 7.20 (t, 1H, 4^1^H), 7.53 (d, 1H, 3^1^H), 7.67 (d, 1H, 5^1^H), 12.8 (s, 1H, NH). Anal. calcd for C_13_H_12_N_4_OS_2_,% N 18.4; S 21.1. Found, % N 18.4; S 21.0.

### 7-(Furan-2-yl)-2-(piperidin-1-yl)[1,3]thiazolo[4,5-d]pyridazin-4(5H)-one (15)

Yield 87%, mp 292–293°C, ^1^H-NMR (400 MHz, DMSO-d_6_), δ: 1.65-1.69 (m, 6H, -CH_2_-CH_2_-CH_2_-), 3.65 (m, 4H, -CH_2_NCH_2_-), 6.70 (t, 1H, 4^1^H), 6.96 (d, 1H, 3^1^H), 7.88 (d, 1H, 5^1^H), 12.9 (s, 1H, NH). ^13^C-NMR (100 MHz, DMSO-d_6_) δ: ^13^C-NMR (100 MHz, DMSO-d_6_) δ: 23.4, 24.8, 49.4, 108.2, 112.4, 124.0, 132.7, 144.0, 148.3, 148.8, 155.1, 171.7. Anal. calcd for C_14_H_14_N_4_O_2_S, % N 18.5; S 10.6. Found, % N 18.4; S 10.7. MS m/z: 303.0 [(M+H)+].

### 7-(Furan-2-yl)-2-(morpholin-4-yl)[1,3]thiazolo[4,5-d]pyridazin-4(5H)-one (16a)

Yield 83%, mp 298–299°C, ^1^HNMR (400 MHz, DMSO-d_6_), δ: 3.65 (t, 4H, -CH_2_NCH_2_-), 3.76 (t, 4H, -CH_2_OCH_2_-), 6.72 (m, 1H, 4^1^H), 6.98 (d, 1H, 3^1^H), 7.91 (d, 1H, 5^1^H), 13.0 (s, 1H, NH). ^13^C-NMR (100 MHz, DMSO-d_6_) δ: ^13^C-NMR (100 MHz, DMSO-d_6_) δ: 48.2, 48.3, 65.3, 108.3, 112.4, 124.4, 132.6, 144.1, 144.1, 148.3, 155.1, 172.3. Anal. calcd for C_13_H_12_N_4_O_3_S, % N 18.4; S 10.5. Found, % N 18.4; S 10.3. MS m/z: 305.1 [(M+H)+].

### 2-(Morpholin-4-yl)-7-(thiophen-2-yl)[1,3]thiazolo[4,5-d]pyridazin-4(5H)-one (16b)

Yield 80%, mp 281–283°C, ^1^H-NMR (400 MHz, DMSO-d_6_), δ: 3.64 (m, 4H, -CH_2_NCH_2_-), 3.76 (m, 4H, -CH_2_OCH_2_-), 7.21 (dd, 1H, 4^1^H), 7.51 (d, 1H, 3^1^H), 7.72 (d, 1H, 5^1^H), 13.0 (c, 1H NH). ^13^C-NMR (100 MHz, DMSO-d_6_) δ: 48.3, 48.4, 65.2, 125.7, 128.0, 128.3, 135.7, 138.6, 148.2, 155.0, 171.4. Anal. calcd for C_13_H_12_N_4_O_2_S_2_, % N 17.5; S 20.0. Found, % N 17.4; S 20.2. MS m/z: 321.0 [(M+H)+].

### Pharmacology

#### Animals

Female non-linear mice (18–22 g) were used. The animals were housed in a quarantine facility for 7 days before the experiment was started. Throughout the experiment, the animals were randomised in groups of four per cage with bedding composed of wood shavings (exchanged daily). The animals had free access to a standard commercial diet and water. The animals were kept under a stable regimen of 12 h light/12 h darkness. All studies were performed under the requirements HEC of the Ministry of Health of Ukraine and the rules of the “European Convention for the Protection of Vertebrate Animals are used with Experimental and other Scientific Purposes” (Strasbourg, 1986).

#### Substances

Ketorolac tromethamine (ketorolac) substance (JSC “Lek-Chem”, Ukraine), carrageenan (Sigma-Aldrich, USA), and acetic acid (“Khimlaborreaktiv”, Ukraine) were used in this study.

The study substance was administered once orally (p.o) at the dose 25 mg/kg [16] in the form of an aqueous-ethanol emulsion using Twin-80 as an emulgator. Ketorolac was administered once orally (p.o.) in the form of an aqueous solution. The dose of 25 mg/kg was selected according to methods described in [17, 18] for it will definitely be considered from the standpoint of the studied compound’s therapeutic impact.

### Analgesia

#### Hot Plate Test

Evaluation of analgesic activity in the experiment was carried out on thermal models of the nociceptive-stimulating “hot plate” [19]. The analgesic activity founded on the change of the latency of “paw licking” (hot plate) was evaluated. At the same time, we determined the percentage of change of the latent period of the reaction relative to the threshold of the reaction at the initial (point). The hot plate test was assessed on groups of five mice. The temperature of the metal surface was maintained at (55 ± 0.2)°C. Latency to a discomfort reaction (licking paws or jumping) was determined before and after drug administration. The cutoff time was 20 sec. The latency was recorded before and 1 hour following p.o. administration of the agents (ketorolac or study substance). The prolongation of the latency times compared with the values of the initial was used for statistical comparison.

#### Acetic Acid-Induced Writhing Model in Mice

The study substance was assessed by the reduction of the number of writhes induced by intraperitoneal injection of 0.6% acetic acid. The number of writhes per animal was counted for 10 min [20, 21]. Inhibition of writhing was calculated by the formula below and compared with the standard drug (ketorolac):

Inhibition of writhing, % = {(Wc − Wt) × 100%}/Wc

where, Wc = number of writhing in the control group; Wt = number of writhing of the experimental group.

### Anti-Inflammatory Activity

#### Carrageenan-Induced Inflammation in Mice

The study substance was administrated p.o. to mice 1 h before subplantar injection of 0.05 ml of 1% carrageenan [22]. The experimental group size was five animals. Three h after carrageenan injection, the mice were withdrawn from the experiment. Hind paws with swollen and non-swollen feet were amputated at the level of the hip joints. The control group was treated with a solvent. The control group size was five animals. The inhibition of edema was calculated using the formula:

Inhibition of edema % = (M_se_ − M_he_)/(M_sc_ − M_hc_) *100% − 100%

where, M_se_=mass of a swollen foot in the experiment; M_he_=mass of a healthy foot in the experiment; M_sc_= mass of a swollen foot in the control; M_hc_= mass of a healthy foot in the control.

Inhibition of edema was calculated by the formula above and compared with the standard drug (ketorolac).

### Statistics

The results were analyzed for statistical significance using variational statistics (t-test) with OriginPro 8.0 software (originLab Corporation, USA) [23].

## Authors’ Statements

### Competing Interests

The authors declare no conflict of interest.

### Animal Rights

All studies were performed in accordance with the requirements of the State Expert Center of the Ministry of Health of Ukraine and the rules of the “European Convention for the Protection of Vertebrate Animals used for Experimental and Other Scientific Purposes” (Strasbourg city, 1986). Animals were sacrificed by decapitation under ether anaesthesia.

## References

[1] Kalkhambkar RG, Kulkarni GM, Shivkumar HR, Nagendra R (2007). Synthesis of novel triheterocyclic thiazoles as anti-inflammatory and analgesic agents. Eur J Med Chem.

[2] Abouzid K, Bekhit SA (2008). Novel anti-inflammatory agents based on pyridazinone scaffold;design, synthesis and in vivo activity. Bioorg Med Chem.

[3] Yokoyama M, Ikuma T, Obara N, Togo H (1990). Synthesis of mesoionic triazoline nucleosides. J Chem Soc Perkin Trans 1.

[4] Gensler WJ, Walter J, Gensler SC, David BB (1981). Synthesis of a triazole homo-C-nucleoside. J Org Chem.

[5] Tamura Y, Adachi M, Kawasaki T, Kita Y (1978). New method for converting amines into 1,1-disubstituted thioureas by using a combined reagent of triphenylphosphine and thiocyanogen. Tetrahedron Lett.

[6] Townley PC, Domagala JM, Peterson P, Bongers S, Nichols JB (1987). New 7-substituted quinolone antibacterial agents. The synthesis of 1-ethyl-1,4-dihydro-4-oxo-7-(2-thiazolyl and 4-thiazolyl)-3-quinolinecarboxylic acids. J Heterocycl Chem.

[7] Abdellatif KR, Chowdhury MA, Dong D, Knaus EE (2008). Diazen-1-ium-1,2-diolated nitric oxide donor ester prodrugs of 1-(4-methanesulfonylphenyl)-5-aryl-1H-pyrazol-3-carboxylic acids: Synthesis, nitric oxide release studies and anti-inflammatory activities.Bioorg Med Chem. http://dx.doi.org/10.1016/j.bmc.2008.05.028.

[8] Burch HA, Gray JE (1972). Acylpyruvates as potential antifungal agents. J Med Chem.

[9] Hoveyda H, Roy M-O, Fraser GL, Dutheuil G Novel nk-3 receptor selective antagonist compounds, pharmaceutical composition and methods for use in nk-3 receptors mediated disorders. Patent.

[10] Quan ML, Wexler RR Substituted amino methyl factor XA inhibitors / M. L. Quan, R. R. Wexler;Bristol-myers squibb company. Patent.

[11] Volochnyuk DM, Ryabukhin SV, Plaskon AS, Dmytriv YV, Grygorenko OO, Mykhailiuk PK, Krotko DG, Pushechnikov A, Tolmachev AA (2010). Approach to the Library of Fused Pyridine-4-carboxylic Acids by Combes-Type Reaction of Acyl Pyruvates and Electron-Rich Amino Heterocycles. J Comb Chem.

[12] Chen YF, Jobanputra P, Barton P, Bryan S (2008). Cyclooxygenase-2 selective non-steroidal anti-inflammatory drugs (etodolac, meloxicam, celecoxib, rofecoxib, etoricoxib, valdecoxib and lumiracoxib) for osteoarthritis and rheumatoid arthritis: a systematic review and economic evaluation. Health Technol Assess.

[13] Berge O-G (2011). Predictive validity of behavioural animal models for chronic pain. Br J Pharmacol.

[14] Deraedt R, Jougney S, Delevalcee F, Falhout M (1980). Release of prostaglandin E and F in an algogenic reaction and its inhibition. Eur J Pharmacol.

[15] Amico-Roxas M, Caruso A, Trombadore S (1984). Gangliosides antinociceptive effects in rodents. Arch Int Pharmacodyn Ther.

[16] Vigorita MG, Ottanà R, Monforte F, Maccari R, Trovato A, Monforte MT, Taviano MF (2001). Synthesis and antiinflammatory, analgesic activity of 3,3′-(1,2-Ethanediyl)-bis[2-aryl-4-thiazolidinone] chiral compounds. Part 10.Bioorg Med Chem Lett.

[17] Stefanov OV (2001). Preclinical studies of drugs (guidelines). Kiev Avicena.

[18] Turner RA (1965). Screening methods in pharmacology.

[19] D'Amour FE, Smith DL (1941). A method for determining lose of pain sensation. J Pharmacol Exp Ther.

[20] Le Bars D, Gozariu M, Cadden SW (2001). Animal Models of Nociception. Pharmacol Rev.

[21] Wood RL, Kuhar M (1941). Animal models in analgesic testing. Analgesics: Neurochemical, Behavioral and Clinical PerspectivesBook. J. Pasternak.

[22] Sluka KA, Westlund KN (1993). Behavioral and immunohistochemical changes in an experimental arthritis model in rats. Pain.

[23] OriginLab®Data Analysis and Graphiny Software (2012). http://www.originlab.com/.

